# Supplementary material for: Immune-profiling of ZIKV-infected patients identifies a distinct function of plasmacytoid dendritic cells for immune cross-regulation
Source: Nat Commun. 2020 May 15;11:2421. doi: 10.1038/s41467-020-16217-5 (PMC7229207; doi:10.1038/s41467-020-16217-5)
Supplement: Supplementary file 1 — Supplementary Informations [file 41467_2020_16217_MOESM1_ESM.pdf]

Supplementary Information

Immune-profiling of ZIKV-infected patients identifies a distinct function of plasmacytoid dendritic cells for immune cross-regulation

Xiaoming Sun, Stephane Hua, Ce Gao, Jane E. Blackmer, Zhengyu Ouyang, Kevin Ard, Andrea Ciaranello, Sigal Yawetz, Paul E. Sax, Eric S. Rosenberg, Mathias Lichterfeld, Xu G. Yu

Supplementary Figure 1

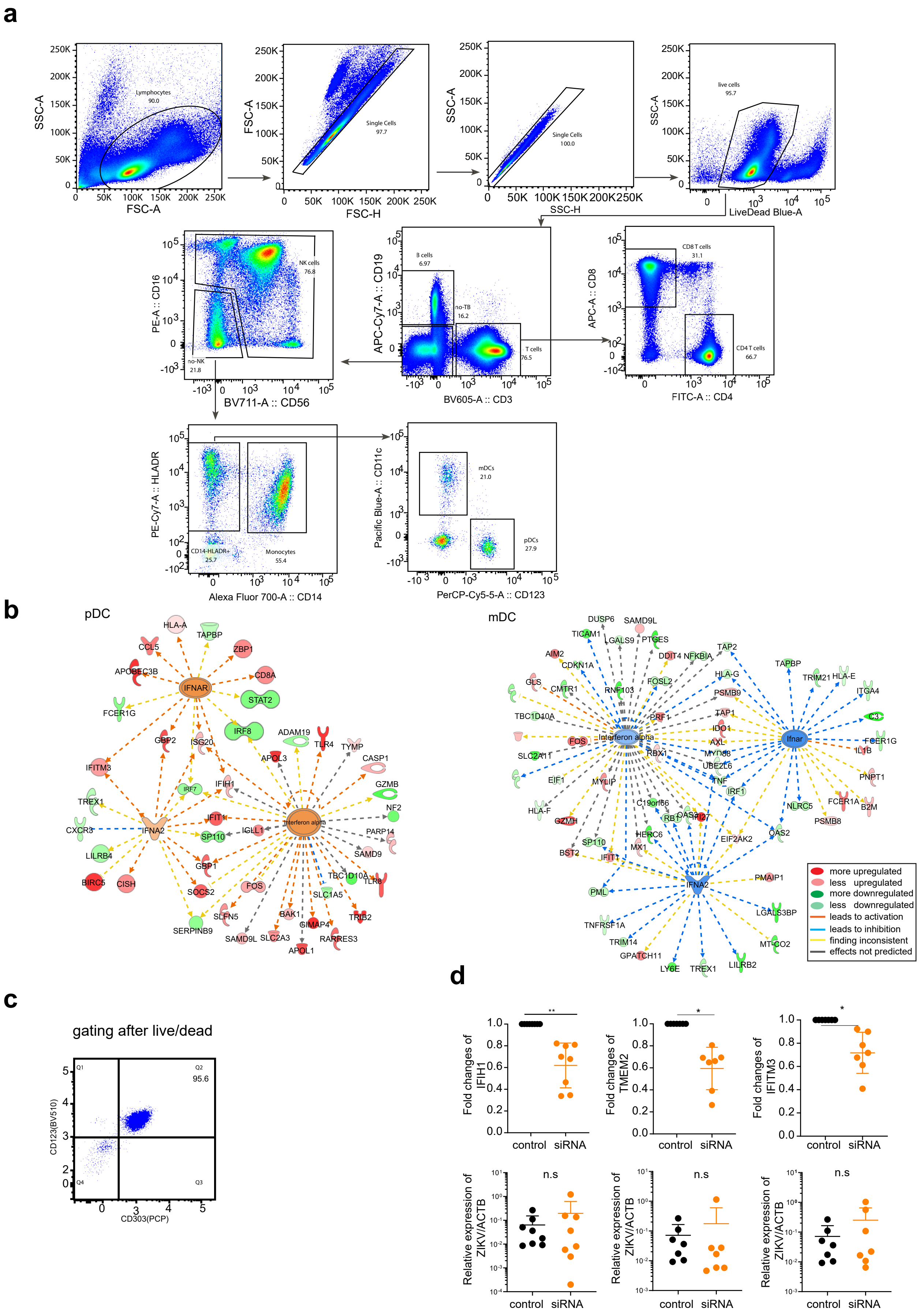

Supplementary Figure 1: a. Gating strategy for CD4 T cells, CD8 T cells, NK cells, monocytes, B cells and DCs from ZIKV-infected and non-infected individuals. **b.** Networks representing upstream regulators of DEGs in pDCs (left panel) and mDCs (right panel) from ZIKV-infected patients predicted to be either activated (orange) or inhibited (blue), as determined by biocomputational analysis using IPA. Symbols of target genes are shown in legend on the right lower panel. **c.** Representative flow cytometry dot plot representing the purity of pDCs after immunomagnetic purification. **d.** mRNA expression of IFIH1, TMEM2 and IFITM3 in pDCs after siRNA-mediated silencing, relative to ACTB (upper panel). Relative ZIKV RNA levels in pDCs after siRNA-mediated silencing of indicated transcripts are shown in lower panels (n=7 biologically independent samples). Horizontal bars reflect the Mean ± SD of biologically independent samples. Statistical significance between the different subsets was tested using two-sided Wilcoxon matched-pairs signed-rank tests. n.s: not significant; *: p<0.05, **: p<0.01.

Supplementary Figure 2

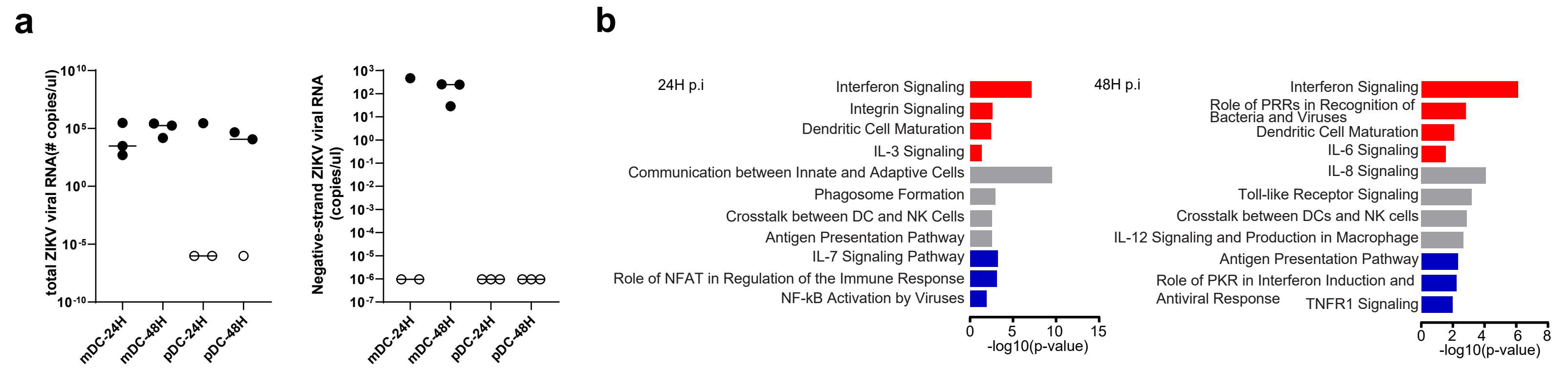

c

pDC(in vitro)

Rank	GeneID	GO term(BP)	Pathway(KEGG)	Pathway(Reactome)
1	CDK1	Positive regulation of response to stimulus(GO:0048584)	EBV infection(hsa05169)	MAPK1/MAPK3 signaling (R-HSA-5684996)
2	JUN	Regulation of immune response (GO:0050776)	NOD-like receptor signaling pathway(hsa04064)	Toll-like receptors cascades (R-HSA-168898)
3	MAPK11	Regulation of cytokine production(GO:0001817)	Toll-like receptor signaling pathway(hsa04620)	Signaling to ERKs (R-HSA-187687)
4	MAPK13	Regulation of cytokine production(GO:0001817)	Toll-like receptor signaling pathway(hsa04620)	Signaling to ERKs (R-HSA-187687)
5	TOP2A	Immune system development (GO:0002520)	-	-
6	STAT1	Regulation of immune process (GO:0002682)	Toll-like receptor signaling pathway(hsa04620)	Interferon alpha/beta signaling (R-HSA-909733)
7	UBD	Defense response (GO:0006952)	-	-
8	AURKB	Regulation of intracellular signal transduction(GO:1902531)	-	-
9	ZAP70	Regulation of immune process (GO:0002682)	Th cell differentitaion (hsa04658)	Adaptive immune system (R-HSA-1280218)
10	NOTCH4	Immune system development (GO:0002520)	-	-

pDC(ex vivo)

Rank	GeneID	GO term(BP)	Pathway(KEGG)	Pathway(Reactome)
1	JUN	Regulation of immune response (GO:0050776)	NOD-like receptor signaling pathway(hsa04621)	Toll-Like Receptors Cascades (R-HSA-168898)
2	LCK	Innate immune response (GO:0045087)	NF-kappa B signaling pathway (hsa04064)	Cytokine Signaling (R-HSA-1280215)
3	AR	Apoptotic process (GO:0006915)	-	Signaling by Rho GTPases (R-HSA-194315)
4	FOS	Regulation of immune response (GO:0050776)	Apoptosis (hsa04210)	Toll-Like Receptors Cascades (R-HSA-168898)
5	UBE2D1	Regulation of immune system process(GO:0002682)	Protein processing in ER (hsa04141)	Toll-Like Receptors Cascades (R-HSA-168898)
6	ACTB	Regulation of immune response (GO:0050776)	Phagosome (hsa04145)	Cytokine Signaling (R-HSA-1280215)
7	YWHAB	Apoptotic process (GO:0006915)	PI3K-Akt signaling pathway (hsa04151)	Cytokine Signaling (R-HSA-1280215
8	JAK3	Apoptotic process (GO:0006915)	-	-
9	IGF1R	Apoptotic process (GO:0006915)	AMPK signaling pathway (hsa04152)	-
10	RANBP2	Protein transport (GO:0015031)	-	Interferon Signaling (R-HSA-913531)

mDC(in vitro)

Rank	GeneID	GO term(BP)	Pathway(KEGG)	Pathway(Reactome)
1	POTEJ	-	-	-
2	ISG15	type I interferon signaling (GO:0060337)	RIG-I like receptor signaling (hsa04622)	Interferon alpha/beta signaling (R-HSA-909733)
3	OAS3	type I interferon signaling (GO:0060337)	NOD-like receptor signaling (hsa04621)	Interferon alpha/beta signaling (R-HSA-909733)
4	OASL	Response to type I interferon (GO:0034340)	-	Interferon alpha/beta signaling (R-HSA-909733)
5	TUBB2A	-	Phagosome (hsa04145)	trafficking to plasma membrane (R-HSA-190841)
6	TUBB4A	-	Phagosome (hsa04145)	trafficking to plasma membrane (R-HSA-190841)
7	TUBA1B	response to cytokine (GO:0034097)	Phagosome (hsa04145)	trafficking to plasma membrane (R-HSA-190841)
8	NOTCH14	Notch receptor processing (GO:0007220)	-	signaling by NOTCH4 (R-HSA-1980150)
9	FBXW7	maintenance of location (GO:0051235)	Ubiquiton mediated proteolysis (hsa04120)	signaling by NOTCH4 (R-HSA-1980150)
10	MAP3K2	Immune system development (GO:0002520)	gap junction (hsa04540)	MAPK1/MAPK3 signaling (R-HSA-5684996)

mDC(ex vivo)

Rank	GeneID	GO term(BP)	Pathway(KEGG)	Pathway(Reactome)
1	SRC	Regulation of ACT (GO:0050776)	regulation of actin cytoskeleton (hsa04810)	Membrane trafficking (R-HSA-199991)
2	RHOA	Innate immune response (GO:0045087)	Endocytosis (hsa04142)	Innate immune system (R-HSA-168249)
3	CUL1	Apoptotic process (GO:0006915)	Ubiquiton mediated proteolysis (hsa04120)	Innate immune system (R-HSA-168249)
4	PPP2CA	Regulation of immune response (GO:0050776)	mRNA surveillance pathway (hsa03015)	Innate immune system (R-HSA-168249)
5	RPL7	Regulation of immune system process(GO:0002682)	Ribosome (hsa03010)	Metabolism of proteins (R-HSA-392499)
6	JUN	Regulation of immune response (GO:0050776)	NOD-like receptor signaling (hsa04621)	Toll-Like Receptors Cascades (R-HSA-168898)
7	LYN	Apoptotic process (GO:0006915)	NFkB signaling pathway (hsa04064)	signaling by interleukins (R-HSA-449147)
8	STAT3	Apoptotic process (GO:0006915)	HIF-1 signaling pathway (hsa04066)	signaling by interleukins (R-HSA-449147)
9	RPL3	Apoptotic process (GO:0006915)	Ribosome (hsa03010)	Metabolism of proteins (R-HSA-392499)
10	GRB2	Protein transport (GO:0015031)	viral carcinogenesis (hsa05203)	signaling by interleukins (R-HSA-449147)

Supplementary Figure 2: a. Total ZIKV RNA and negative strand ZIKV RNA in pDCs and mDCs sorted from PBMCs at indicated time points after in vitro infection (n=3 biologically independent samples). Horizontal lines reflect the Median values of three independent samples. **b.** "Canonical pathways" inferred by IPA from DEGs between in vitro ZIKV-infected pDCs and control pDCs at 24h and 48h post infection. Red and blue color coding denotes functional pathways predicted to be up- or downregulated, respectively; grey indicates indeterminate directional changes for the respective functional entity. **c.** Tables indicating the predicted functions of the hub genes of pDCs and mDCs shown in figure 4c, based on transcriptional profiling data collected from ZIKV-infected patients (in vivo) or in vitro-infected cells (in vitro). Analysis was performed using Gene ontology analysis, KEGG, and Reactome pathways analysis.

Supplementary Figure 3

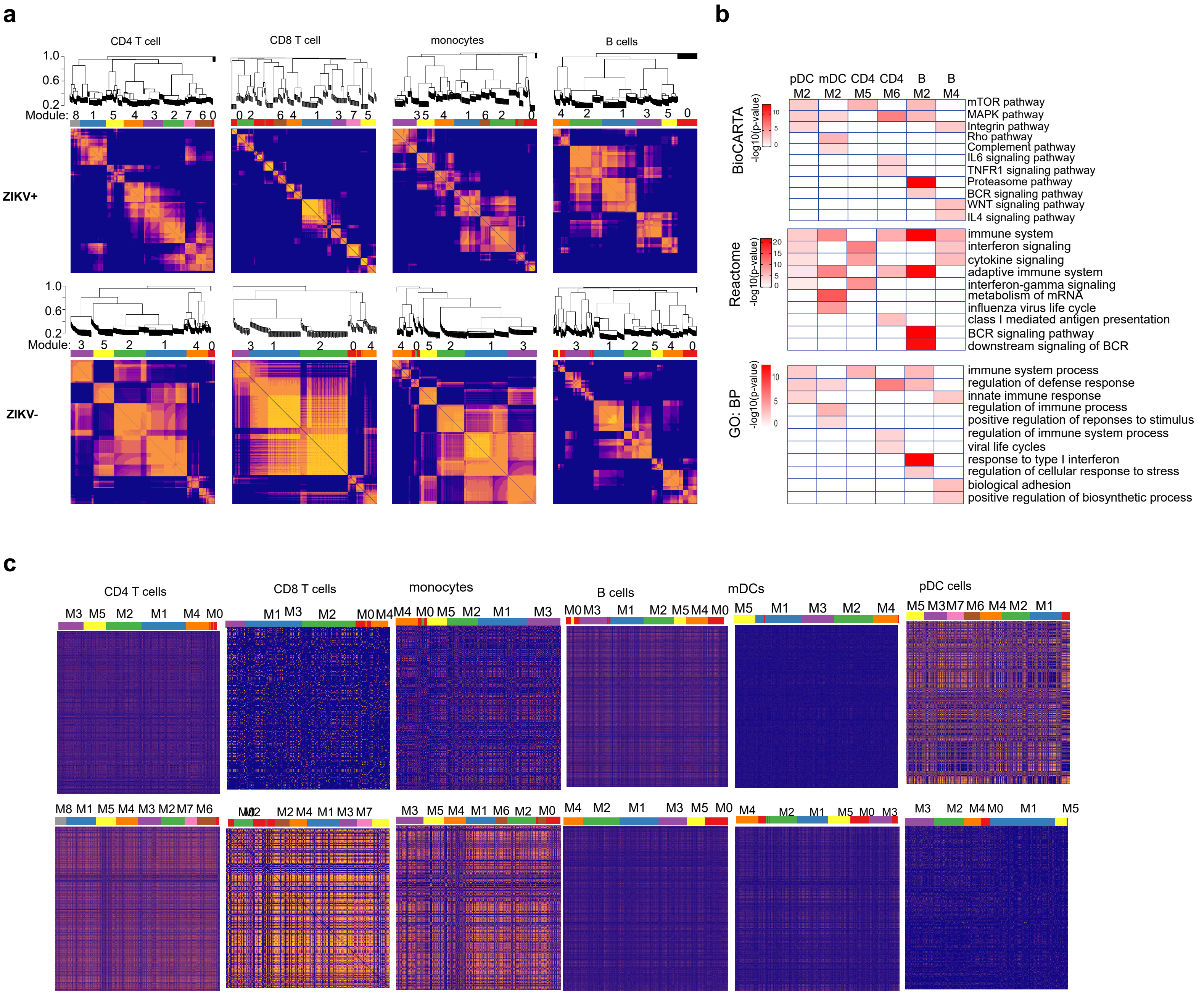

Supplementary Figure 3: a Heatmaps highlighting modules of transcripts with correlated gene expression patterns in indicated immune cells subsets from ZIKV-infected and non-infected individuals. **b.** Heatmaps highlighting the pathways that genes from each analyzed module were enriched for, as determined by BioCARTA, Reactome, and Gene ontology analysis **c.** Heatmaps highlighting transcription modules of ZIKV-infected patients applied to immune cell subsets from non-infected individuals (upper panel) and transcriptional modules of non-infected individuals applied to immune cell subsets of ZIKV-infected patients (lower panel).

Supplementary Figure 4

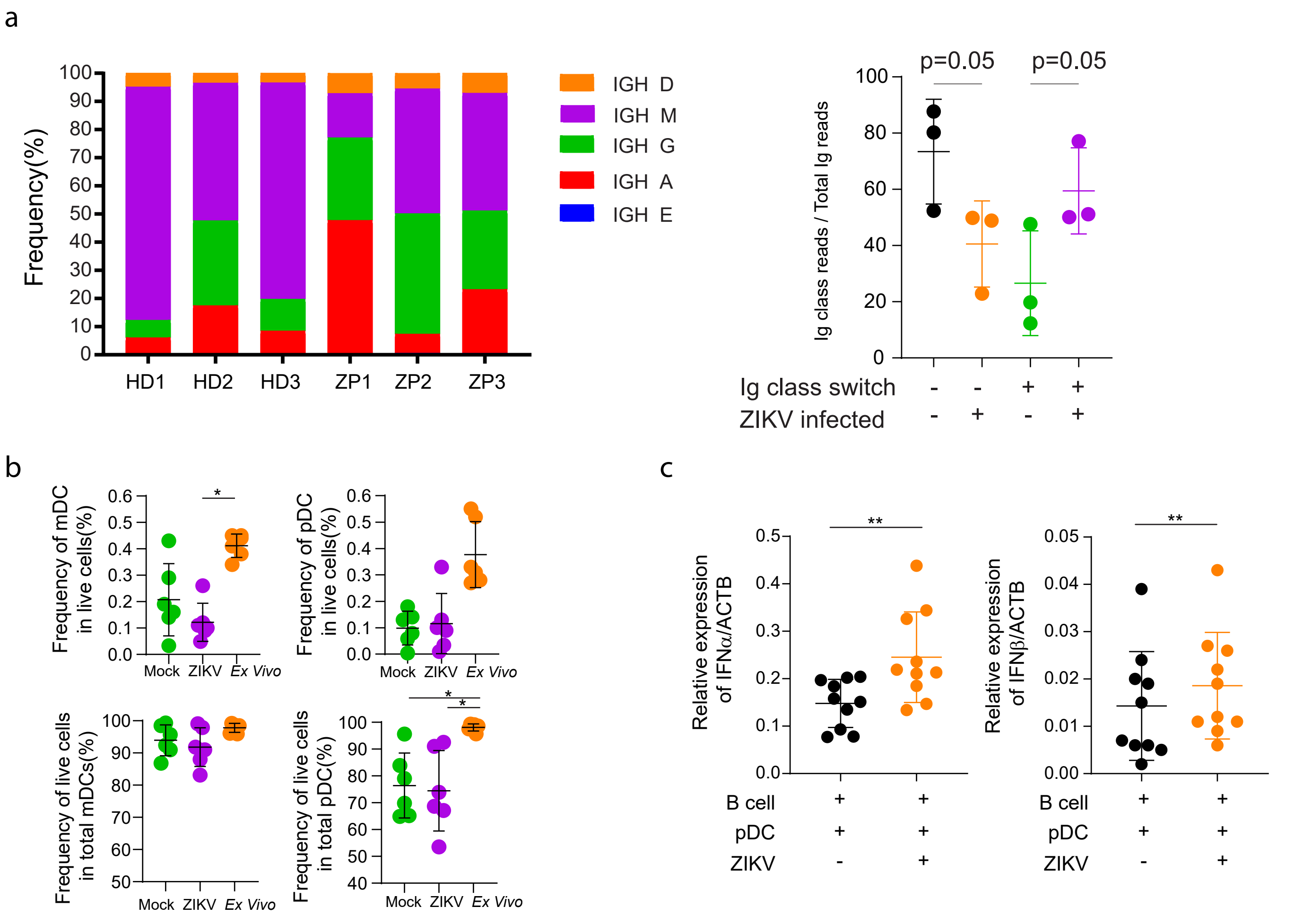

Supplementary Figure 4: **a.** (left panel) Bar diagrams reflecting the expression intensity of transcripts encoding the heavy chains of different immunoglobulins in B cells (n= 3 biologically independent samples from healthy controls and n=3 biologically independent samples from ZIKV-infected patients). (right panel) Single data plots reflecting the expression intensity of indicated Ig-encoding transcripts relative to total immunoglobulin transcripts in uninfected control individuals and ZIKV-infected patients. Ig classes with (IgG, IgA, IgE) and without (IgM, IgD) class switching are indicated. **b.** Cell viability and proportions of mDCs and pDCs in in vitro cultured PBMC with or without exposure to ZIKV infection (n=6 biologically independent samples). Data from ex vivo isolated pDC and mDC are shown for comparison. Horizontal bars reflect Mean ± SD. c. Expression of IFN- or - relative to -actin mRNA, measured in pDC after 24 hours of co-culture with B cells. Data from ZIKV-infected and uninfected samples are shown. (n=10 biologically independent samples). Statistical significance between the different subsets was tested using two-sided Wilcoxon matched-pairs signed-rank tests. *: p<0.05, **: p<0.01

Supplementary Figure 5

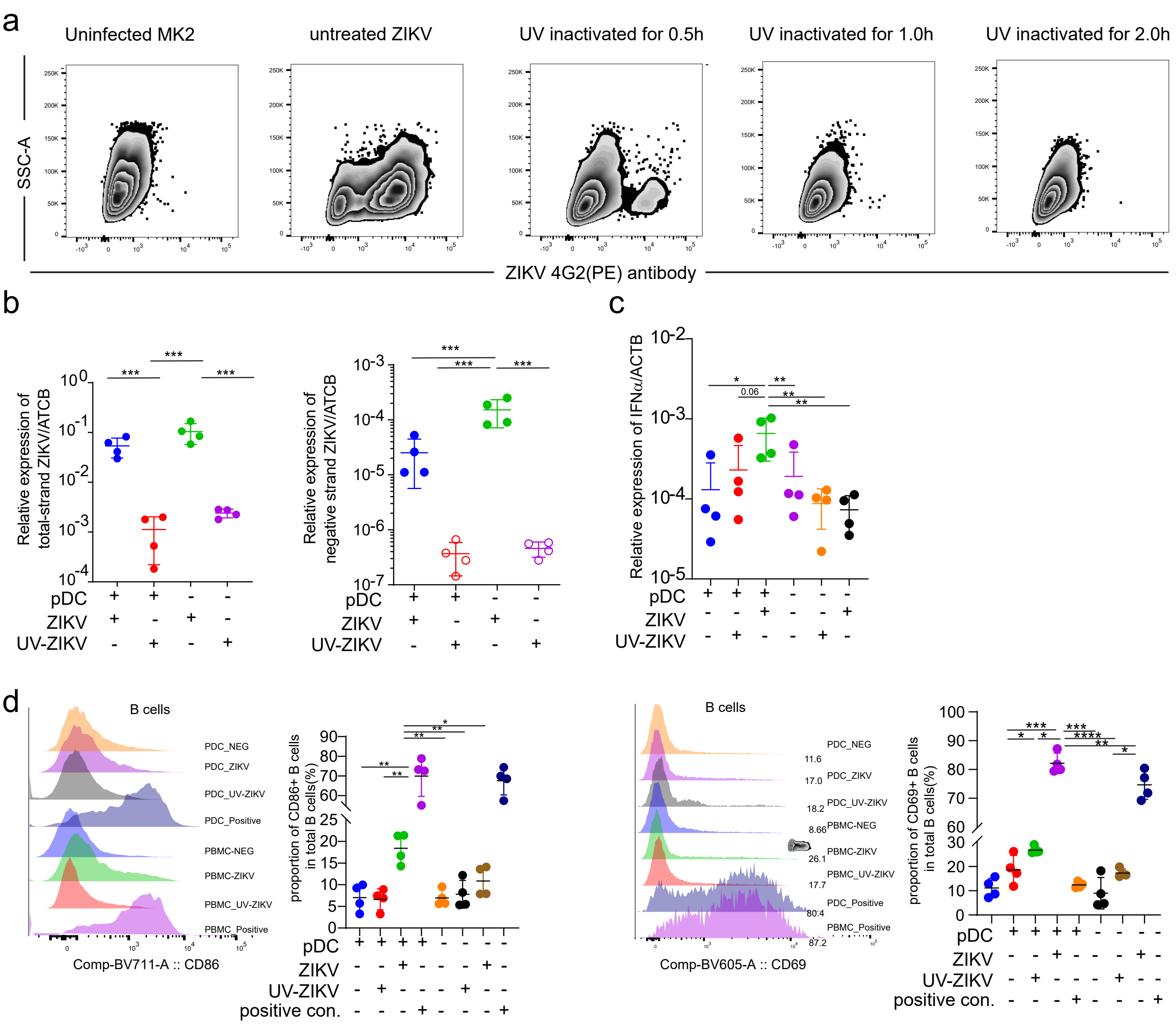

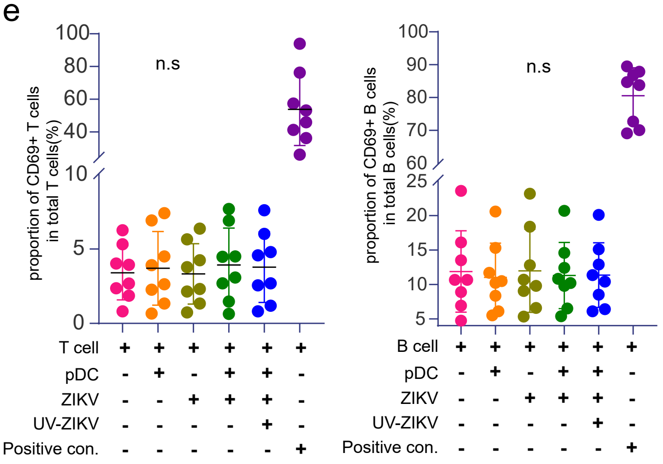

Supplementary Figure 5: a. Flow cytometric detection of ZIKV glycoprotein 4G2 in MK2 cells following exposure to replication-competent (“untreated”) or UV-inactivated ZIKV. The ZIKV stock was treated with UV light (wavelength of 250-270 nm) for 30, 60 or 120 minutes at room temperature and used to infect MK2 cells; cells were analyzed 24 hours post-infection. b. ZIKV replication in PBMC with or without depletion of pDCs. Total PBMCs or pDC-depleted PBMCs were infected with ZIKV or UV-inactivated ZIKV for 24 hours (n=4 biologically independent samples). Total and negative-stranded ZIKV RNA was measured by qPCR. Open circle indicated limit of detection. c. IFN- mRNA expression in total or pDC-depleted PBMCs at 24 hours after exposure to replication-competent or UV-inactivated ZIKV. IFN- mRNA was measured by qPCR (n=4 biologically independent samples). d. Expression of CD86 and CD69 on B cells within total or pDC-depleted PBMCs, at 24 hours following exposure to replication-competent or UV-inactivated ZIKV (n=4 biologically independent samples). e. Expression of CD69 on purified B cells or T cells (1×105/well) after co-culture with pDCs (0.4×105/well) in separated compartments using a transwell system in the presence or absence of replication-competent or UV-inactivated ZIKV or positive control for 24 h (n=8 biologically independent samples) are presented as Mean value ±SD. Statistical significance between the different subsets was tested using a Friedman test with post-hoc Dunn’s test. n.s: not significant; *: $p<0.05$, **: $p<0.01$, ***: $p<0.001$, ****: $p<0.0001$

Supplemental Table 1: clinical information of ZIKV-infected patients

Parameters	Patient A	Patient B	Patient C	Healthy donors
**Age**	35	32	31	28-33 (Median 31, n=3)
**Gender**	Female	Female	Female	Female
**Pregnancy**	no	no	no	no
**Traveling history**	St. Vincent, Cayman	Puerto Rico	Santo Domingo	-
**Fever**	+	+	+	-
**Rash**	+	+	+	-
**Joint pain**	+	+	+	-
**Other symptom**	-	flu-like, cough, diarrhea, fatigue	ankle edema	-
**Dengue virus PCR test**	-	-	-	n.t
**Chikungunya virus PCR test**	-	-	-	n.t

Supplementary Table 2： Sequences of primers used in this study

EMC 7 primers

F: CAAGTGATCCTGACATGAGACGG

R: TGCCGCTGCTAGATTTGCCAGA

TMEM2 primers

F: GGAATAGGACTGACCTTTGCCAG

R: TTCTGACCACCCTGAAAGCCGT

IDO1 primers

F: GCCTGATCTCATAGAGTCTGGC

R: TGCATCCCAGAACTAGACGTGC

IFIH1 primers

F: GCTGAAGTAGGAGTCAAAGCCC

R: CCACTGTGGTAGCGATAAGCAG

IFITM3 primers

F: CTGGGCTTCATAGCATTCGCCT

R: AGATGTTCAGGCACTTGGCGGT

ACTB primers

F: CACCATTGGCAATGAGCGGTTC

R: AGGTCTTTGCGGATGTCCACGT

ZIKV Primers

F: CCGCTGCCCAACACAAG

R: CCACTAACGTTCTTTTGCAGACAT

Probe: FAM /56- FAM/AGCCTACCT/ZEN/TGACAAGCAGTCAGACACTCAA/31ABkFQ/
