# Supplementary material for: Immune-profiling of ZIKV-infected patients identifies a distinct function of plasmacytoid dendritic cells for immune cross-regulation
Source: Nat Commun. 2020 May 15;11:2421. doi: 10.1038/s41467-020-16217-5 (PMC7229207; doi:10.1038/s41467-020-16217-5)
Supplement: Supplementary file 3 — Description of Additional Supplementary Information [file 41467_2020_16217_MOESM3_ESM.pdf]

Description of Additional Supplementary Files

File Name: Supplementary Data 1
Description: List of Differentially-Expressed Genes in B cells, CD4 T cells, CD8 T cells, myeloid dendritic cells, monocytes, NK cells and plasmacytoid dendritic cells

File Name: Supplementary Data 2
Description: Canonical Pathways and Diseases and Functions analysis from Ingenuity Pathway Analysis of DEGs of B cells, CD4 T cells, CD8 T cells, myeloid dendritic

File Name: Supplementary Data 3
Description: Comparison of upstream regulators analyzed with Ingenuity Pathway Analysis from B cells, CD4 T cells, CD8 T cells, myeloid dendritic cells, monocytes and plasmacytoid dendritic cells

File Name: Supplementary Data 4
Description: List of Zika virus dependency genes and Interferon stimulated genes in B cells, myeloid dendritic cells, monocytes and plasmacytoid dendritic cells

File Name: Supplementary Data 5
Description: Gene Set Enrichment Analysis of Zika virus dependency genes and Interferon stimulated genes

File Name: Supplementary Data 6
Description: Canonical Pathways and Diseases and Functions analysis from Ingenuity Pathway Analysis of DEGs of myeloid dendritic cells and plasmacytoid dendritic cells ex vivo and in vitro at 24h and 48h post infection

File Name: Supplementary Data 7
Description: List of genes in each module of the WGCNA of B cells, CD4 T cells, myeloid dendritic cells, monocytes and plasmacytoid dendritic cells

File Name: Supplementary Data 8
Description: Canonical Pathways analysis from Ingenuity Pathway Analysis of genes of correlated modules of B cells, CD4 T cells, myeloid dendritic cells, monocytes and plasmacytoid dendritic cells
