# Supplementary material for: Immune-profiling of ZIKV-infected patients identifies a distinct function of plasmacytoid dendritic cells for immune cross-regulation
Source: Nat Commun. 2020 May 15;11:2421. doi: 10.1038/s41467-020-16217-5 (PMC7229207; doi:10.1038/s41467-020-16217-5)
Supplement: Supplementary file 12 — Reporting summary [file 41467_2020_16217_MOESM12_ESM.pdf]

nature research

Corresponding author(s): Xu Yu

Last updated by author(s): Mar 3, 2020

Reporting Summary

Nature Research wishes to improve the reproducibility of the work that we publish. This form provides structure for consistency and transparency in reporting. For further information on Nature Research policies, see Authors & Referees and the Editorial Policy Checklist .

Statistics

For all statistical analyses, confirm that the following items are present in the figure legend, table legend, main text, or Methods section.

n/a	Confirmed	
☐	☒	The exact sample size (*n*) for each experimental group/condition, given as a discrete number and unit of measurement
☐	☒	A statement on whether measurements were taken from distinct samples or whether the same sample was measured repeatedly
☐	☒	The statistical test(s) used AND whether they are one- or two-sided<br>*Only common tests should be described solely by name; describe more complex techniques in the Methods section.*
☒	☐	A description of all covariates tested
☐	☒	A description of any assumptions or corrections, such as tests of normality and adjustment for multiple comparisons
☐	☒	A full description of the statistical parameters including central tendency (e.g. means) or other basic estimates (e.g. regression coefficient) AND variation (e.g. standard deviation) or associated estimates of uncertainty (e.g. confidence intervals)
☐	☒	For null hypothesis testing, the test statistic (e.g. *F*, *t*, *r*) with confidence intervals, effect sizes, degrees of freedom and *P* value noted<br>*Give P values as exact values whenever suitable.*
☒	☐	For Bayesian analysis, information on the choice of priors and Markov chain Monte Carlo settings
☒	☐	For hierarchical and complex designs, identification of the appropriate level for tests and full reporting of outcomes
☒	☐	Estimates of effect sizes (e.g. Cohen's *d*, Pearson's *r*), indicating how they were calculated

Our web collection on statistics for biologists contains articles on many of the points above.

Software and code

Policy information about availability of computer code

Data collection	BD FACSDiva 8.0.1, NextSeq System Suite v2.2.0 and QuantStudio Real Time PCR software v1.3
Data analysis	GraphPad Prism v7, R v3.5.2, Cytoscape v3.7.2, STAR aligner, IPA v49932394, WGCNA, WebGestalt 2019

For manuscripts utilizing custom algorithms or software that are central to the research but not yet described in published literature, software must be made available to editors/reviewers. We strongly encourage code deposition in a community repository (e.g. GitHub). See the Nature Research guidelines for submitting code & software for further information.

Data

Policy information about availability of data

All manuscripts must include a data availability statement. This statement should provide the following information, where applicable:

- Accession codes, unique identifiers, or web links for publicly available datasets
- A list of figures that have associated raw data
- A description of any restrictions on data availability

All data reported in this study are available from the corresponding author upon request. The RNA-seq data reported in this paper have been deposited to the NCBI GEO and are available under accession number GSE132228. Hg38 human genome database and Interferome v2.0 database were used in this study.

Field-specific reporting

Please select the one below that is the best fit for your research. If you are not sure, read the appropriate sections before making your selection.

☒ Life sciences ☐ Behavioural & social sciences ☐ Ecological, evolutionary & environmental sciences

Life sciences study design

All studies must disclose on these points even when the disclosure is negative.

Sample size	Statistical tests were not used to determine group sizes. For RNAseq, 3 healthy donors and 3 ZIKV-infected patients were included in this study and for in vitro experiments, 11 healthy donors were included
Data exclusions	In the context of the gene silencing experiments with siRNA, results from experiments in which gene silencing was unsuccessful were excluded.
Replication	PCR experiments were run in duplicate per our standard protocol.
Randomization	N/A
Blinding	N/A

Reporting for specific materials, systems and methods

We require information from authors about some types of materials, experimental systems and methods used in many studies. Here, indicate whether each material, system or method listed is relevant to your study. If you are not sure if a list item applies to your research, read the appropriate section before selecting a response.

Materials & experimental systems

n/a	Involved in the study	
☐	☒	Antibodies
☐	☒	Eukaryotic cell lines
☒	☐	Palaeontology
☒	☐	Animals and other organisms
☐	☒	Human research participants
☒	☐	Clinical data

Methods

n/a	Involved in the study	
☒	☐	ChIP-seq
☐	☒	Flow cytometry
☒	☐	MRI-based neuroimaging

Antibodies

Antibodies used

Antibody Clone Company Fluorochrome Catalog number
CD3 clone OKT3 Biolegend BV605 317322
CD4 clone OKT4 Biolegend FITC 317408
CD8 clone SK1 Biolegend APC 344722
CD19 clone HIB19 Biolegend APC-Cy7 302218
CD56 clone HCD56 Biolegend BV711 318336
CD16 clone B73.1 Biolegend PE 360704
CD14 clone HCD14 Biolegend Alexa Fluor 700 325614
HLA-DR clone L243 Biolegend PE-Cy7 307616
CD11c clone 3.9 Biolegend BV421 301628
CD123 clone 6H6 Biolegend PerCP-Cy5.5 306016
CD69 clone FN50 Biolegend BV605 310938
CD86 clone IT2.2 Biolegend BV711 305440
CD19 clone HIB19 Biolegend FITC 302206
CD123 clone 6H6 Biolegend BV510 306022
CD303 clone 201A Biolegend PerCP 354210
ZIKV 4G2 (clone D1-4G2-4-15 (4G2)) Novus Biologicals PE NBP2-52709PE

Validation

See Methods section

Eukaryotic cell lines

Policy information about cell lines

Cell line source(s)	Aedes albopictus clone C6/36 was purchased from ATCC (ATCC® CRL-1660™)<br>LLC-MK2 cell line was purchased from ATCC (ATCC® CCL-7™)
Authentication	None of the cell line used were authenticated

Mycoplasma contamination	The cell lines were not tested for mycoplasma contamination
Commonly misidentified lines (See ICLAC register)	*Name any commonly misidentified cell lines used in the study and provide a rationale for their use.*

Human research participants

Policy information about studies involving human research participants

Population characteristics	See Supplemental Table 9
Recruitment	Blood samples were collected from individuals with Zika Virus infection from the Massachusetts General Hospital and the Brigham and Women's Hospital (Boston, MA) (n=3). qRT-PCR confirmed ZIKV-positive patients in this study. Healthy donors were recruited from the Massachusetts General Hospital (Boston, MA) (n=11)
Ethics oversight	IRB of Massachusetts General Hospital, Boston (protocol number: 2016P000319)

Note that full information on the approval of the study protocol must also be provided in the manuscript.

Flow Cytometry

Plots

Confirm that:

- [x] The axis labels state the marker and fluorochrome used (e.g. CD4-FITC).
- [x] The axis scales are clearly visible. Include numbers along axes only for bottom left plot of group (a 'group' is an analysis of identical markers).
- [x] All plots are contour plots with outliers or pseudocolor plots.
- [x] A numerical value for number of cells or percentage (with statistics) is provided.

Methodology

Sample preparation	See Methods section
Instrument	BD FACS Fortessa
Software	FlowJo
Cell population abundance	CD4 T cells: 25%<br>CD8 T cells: 20%<br>B cells: 5%<br>NK: 3%<br>monocytes: 5%<br>mDC: 0.1%<br>pDC: 0.1%<br>purity was assessed by flow cytometry and was greater than 95%.
Gating strategy	See Supplementary Figure 1 and Methods section

- [x] Tick this box to confirm that a figure exemplifying the gating strategy is provided in the Supplementary Information.
